# Treatment options for patients with CADASIL and large-scale cerebral infarction: mechanical thrombectomy and antiplatelet therapy—A case report

**DOI:** 10.3389/fneur.2024.1400537

**Published:** 2024-06-19

**Authors:** Shuyue Xiao, Man Ke, Kaiwei Cai, Anding Xu, Menglong Chen

**Affiliations:** ^1^Department of Neurology and Stroke Center, The First Affiliated Hospital of Jinan University, Guangzhou, China; ^2^Clinical Neuroscience Institute, The First Affiliated Hospital of Jinan University, Guangzhou, China; ^3^Key Lab of Guangzhou Basic and Translational Research of Pan-vascular Diseases, The First Affiliated Hospital of Jinan University, Guangzhou, China

**Keywords:** CADASIL, large-scale cerebral infarction, mechanical thrombectomy, antiplatelet therapy, intracranial non-small vessels

## Abstract

**Background:**

Cerebral autosomal dominant arteriopathy with subcortical infarcts and leukoencephalopathy (CADASIL) is an autosomal dominant inherited arterial disease, with lacunar infarction resulting from intracranial small vessel lesions being the most prevalent clinical manifestation of CADASIL. However, large-scale cerebral infarction caused by intracranial non-small vessels occlusion is relatively uncommon, and reports of vascular intervention and long-term antiplatelet drug treatment for patients with CADASIL and large-scale cerebral infarction are rarer.

**Methods:**

We reported a 52 year-old male who experienced a significant cerebral infarction due to an occlusion in the second segment of the left middle cerebral artery, 4 months subsequent to being diagnosed with CADASIL. Following the benefit and risk assessment, the patient underwent intracranial vascular thrombectomy and balloon dilation angioplasty. Subsequently, he was administered dual antiplatelet therapy for 3 months, followed by mono antiplatelet therapy.

**Results:**

After undergoing intracranial vascular intervention and receiving antiplatelet therapy, significant improvement in the symptoms were observed. The National Institutes of Health Stroke Scale score decreased from 6 to 2 points, and no bleeding lesions were detected on the head computed tomography during regular follow-up visits after discharge.

**Conclusion:**

Our case highlights the possibility that patients with CADASIL may also encounter extensive cerebral infarction resulting from stenosis or occlusion of intracranial non-small vessels. Considering the specific circumstances of the patient, intravascular intervention and antiplatelet therapy can be regarded as viable treatment options for individuals with CADASIL.

## 1 Introduction

Cerebral autosomal dominant arteriopathy with subcortical infarcts and leukoencephalopathy (CADASIL) is a monogenic hereditary small vessel disease caused by a mutation in the *NOTCH3* gene located at 19p13.2-p13.1 (1). The receptor encoded by *NOTCH3* is highly expressed in vascular smooth muscle cells (VSMCs) and pericytes (1), primarily affecting small penetrating cerebral and leptomeningeal arteries (2). Genetic testing and the identification of granular osmiophilic material in the vascular wall under an electron microscope are the gold standard criteria for diagnosing CADASIL (3, 4). In terms of imaging, the presence of white matter hyperintensity (WMH) found in magnetic resonance imaging (MRI) is a characteristic feature of CADASIL (5, 6). WMH mainly presents around the ventricles, anterior temporal pole, external capsule, and frontal and parietal lobes (7), with the frontal lobe bearing the highest burden of lesions (5, 8). The involvement of the external capsule and temporal pole strongly suggests CADASIL. Additionally, the presence of lacunar infarct and cerebral microbleed lesions throughout various brain regions may also be observed (9).

Migraine with aura, recurrent ischemic stroke (IS), mental disorders, and cognitive impairment are the four major clinical manifestations of CADASIL (2, 10). Among them, transient ischemic attacks and acute ischemic stroke (AIS) are the most prevalent. Ischemic lesions typically present as lacunar infarctions involving the subcortical white matter (11). Generally, non-small vessels are not affected (12, 13), and large-area cerebral infarctions are even less common. In terms of treatment, further data and research are necessary to determine whether antiplatelet drugs can offer greater benefits to patients with CADASIL with AIS (2). Therefore, uncertainties persist regarding the use of antiplatelet drugs for patients with CADASIL, and there have been no reported cases of endovascular interventional treatment.

## 2 Case description

**Figure 1:**
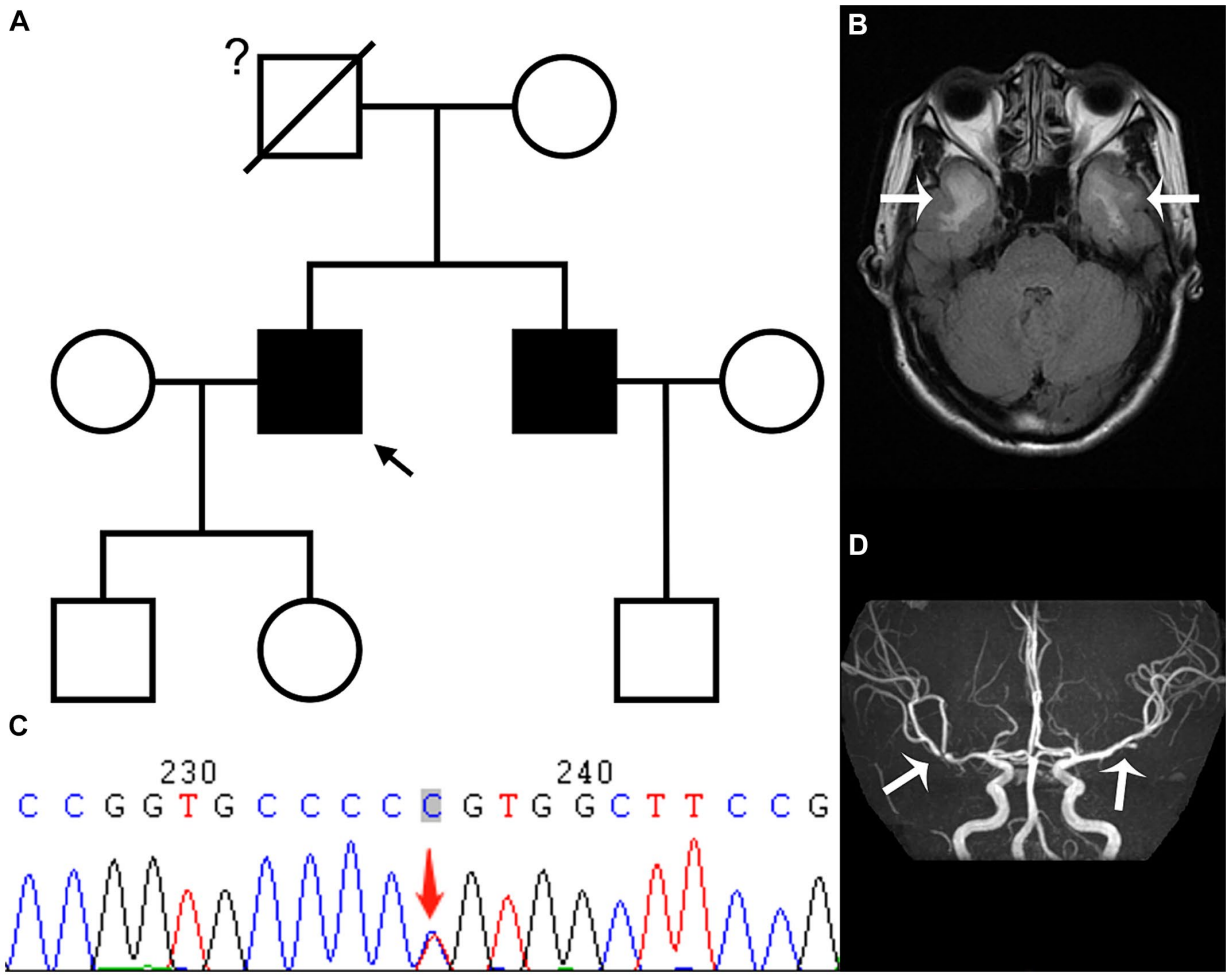
The family tree **(A)** reveals that the proband (black arrow) and his younger brother have CADASIL, while we are uncertain whether the proband’s father had CADASIL; the T2 fluid attenuated inversion recovery sequence **(B)** of the head magnetic resonance imaging in February shows demyelinating lesions in the white matter of bilateral temporal pole (white arrow); while genetic testing **(C)** shows a heterozygous c.328C > T (p.R110C) missense mutation in exon 3 of the *NOTCH3* gene; the magnetic resonance angiography sequence **(D)** indicates local stenosis of the left of the second middle cerebral artery (MCA) segment and the right of the first MCA segment (white arrow).

The proband, a 52 year-old right-handed male, was admitted to the hospital in February 2023 with a 5 year history of memory loss and recurring headaches over the past 6 months. His memory loss primarily affected the recall of recent events, necessitating reminders from others, and he often forgot about performing tasks immediately after completing them. He complained of pulsating headaches at the top and temporal regions. Physical examination indicated memory loss and poor computational ability, but directional ability was normal, and his Montreal Cognitive Assessment (MOCA) score was 20 points. His medical history included a long-standing diagnosis of hepatitis B, with no history of hypertension, coronary heart disease, or diabetes. The patient did have a history of smoking for 1 year, consuming 2–4 cigarettes per week. Notably, the father of the proband had succumbed to AIS approximately 30 years prior. The patient’s younger brother was diagnosed with AIS in October 2019; head MRI using a diffusion-weighted imaging (DWI) sequence revealed a cerebral infarction in the left basal ganglia-corona radiate area (Supplementary Figure S1). Additionally, demyelinating lesions were evident in the white matter, temporal pole, external capsule, central oval, and radiocoronal regions around the bilateral lateral ventricles on a T2 fluid attenuated inversion recovery (FLAIR) sequence (Figure 1A; Supplementary Figure S2). Upon the proband’s admission, laboratory tests indicated a total cholesterol level of 5.20 mmol/L, low-density lipoprotein-C (LDL-C) at 2.89 mmol/L, and glycosylated hemoglobin (HbA1c) of 6.1%. The color Doppler ultrasound of the neck vessels revealed bilateral carotid atherosclerosis (AS). Further evaluation of complete brain T2 FLAIR sequences unveiled multiple demyelinating lesions in the white matter of the bilateral lateral ventricles, central half oval, coronal radiation, bilateral external sacs and bilateral temporal pole (Figure 1B). Additionally, small softening lesions were observed in the right central half oval. Magnetic resonance angiography and transcranial Doppler (TCD) suggested localized stenosis in the left of the second middle cerebral artery (MCA) segment (M2) and the right of the first MCA segment (Figure 1D). Subsequently, genetic testing was conducted in consultation with the proband, revealing a heterozygous c.328C > T (p.R110C) missense mutation in exon 3 of the *NOTCH3* gene (Figure 1C). Genetic testing results, in conjunction with the patient’s clinical symptoms, signs, and imaging findings, led to a diagnosis of CADASIL. During his hospitalization, the proband received donepezil and memantine to enhance cognitive function, nonsteroidal anti-inflammatory drugs for headache management, and rosuvastatin calcium to address the lipid levels. Six days later, the patient felt an improvement in headache symptoms and was subsequently discharged from the hospital.

**Figure 2:**
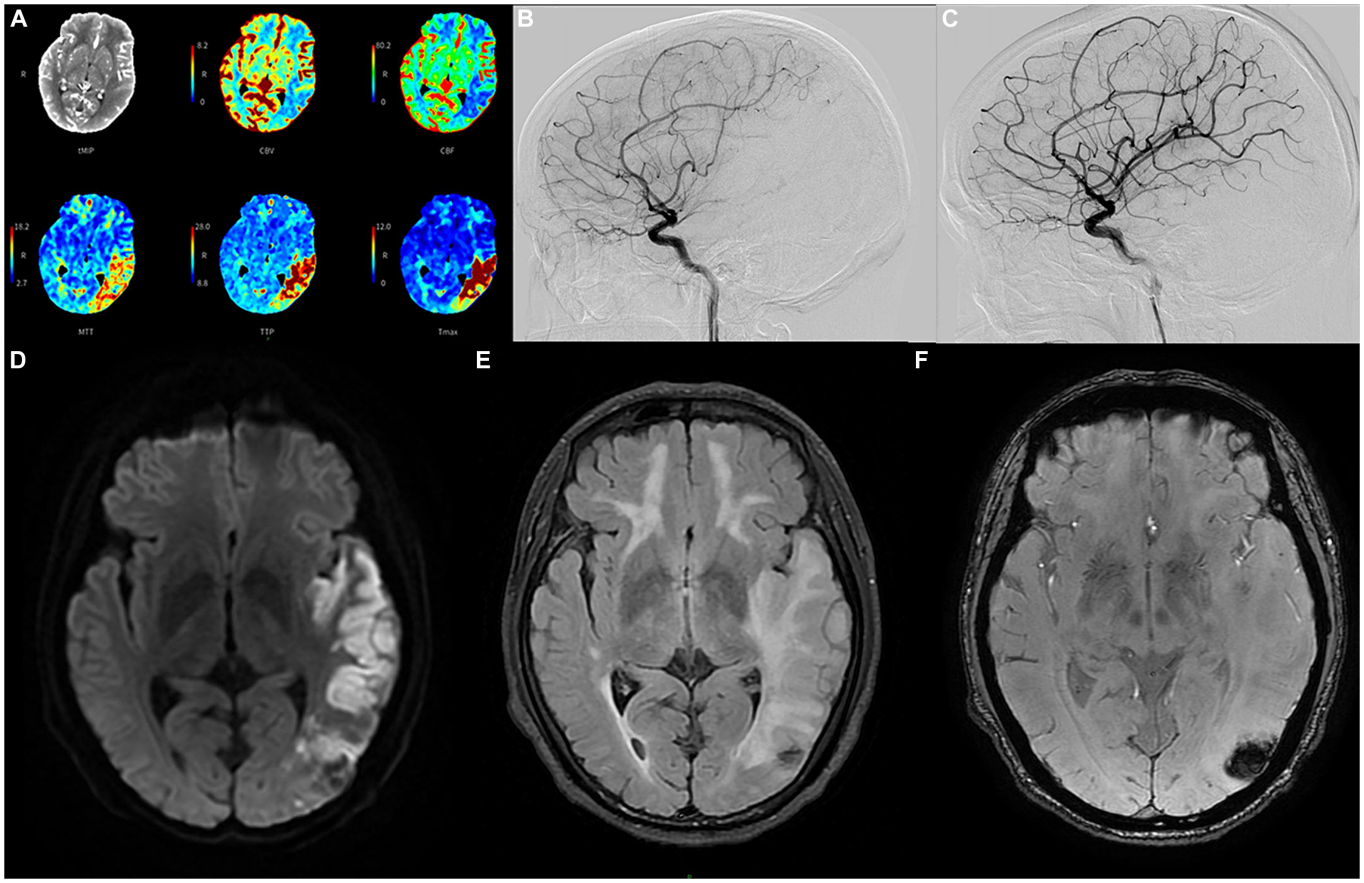
The emergency computer tomography perfusion **(A)** of the proband reveals that the cerebral blood flow and volume in the low-density area of the left temporo-parieto-occipital insular lobe were lower than those on the contralateral side. Additionally, the mean transit time, time to peak, and *T*_max_ were longer than those on the contralateral side; the volume of the core infarct area was 48.6 mL, and the mismatch volume was 41.8 mL. The volume of the perfusion area was 90.4 mL, resulting in a mismatch ratio of 1.9; cerebral angiography conducted before recanalization **(B)** depicts severe stenosis (80%) in the long segment of the initial portion of the inferior trunk of the left middle cerebral artery. Follow-up angiography conducted after endovascular treatment **(C)** demonstrates significant improvement in the stenosis; the diffusion-weighted imaging sequence **(D)** of the head magnetic resonance imaging after angioplasty in June shows cerebral infarction in multiple locations; the T2 fluid attenuated inversion recovery sequence **(E)** shows multiple demyelinating changes in the white matter of the brain; while the T2 star-weighted angiography sequence **(F)** indicates schistose hemorrhagic lesions in the parietal and occipital lobes.

Four months later, the proband experienced a sudden onset of numbness in his right lower limb and slurred speech at 4 a.m. and was admitted to our hospital 5 h later. Upon conducting a neurological examination, it was evident that he maintained clear consciousness but displayed deficits in temporal and spatial orientation. Moreover, there was a noticeable decrease in character orientation, compromised computational abilities, memory impairment, and symptoms of speech ambiguity. Notably, he presented mixed and naming aphasia. The right lower limb muscle strength was graded at V-, accompanied by diminished sensation and a positive right Babinski sign. A prompt assessment of stroke-related criteria assigned the proband a National Institutes of Health Stroke Scale (NIHSS) score of 6 points upon admission. The Modified Rankin Scale (mRS) score was 1 point. Subsequently, an emergency head computed tomography (CT) examination revealed an extensive cerebral infarction in the left temporal parietal occipital lobe (Supplementary Figure S3), the Alberta Stroke Program Early CT Score (ASPECTS) was 8. Further evaluation via computer tomography angiography disclosed occlusion of the distal lumen in the left of the M2 (Supplementary Figure S4). Besides, the computer tomography perfusion (CTP) showed that the volume of the core infarct area was 48.6 mL, the volume of the hypoperfusion area was 90.4 mL, and the mismatch ratio was 1.9 (Figure 2A).

Many studies have found that compared with standard medical treatment, thrombectomy for patients with M2 occlusion has a tendency to improve clinical efficacy (14, 15), and achieving reperfusion is also closely related to good prognosis (16). The proband was within the endovascular treatment time window when he was admitted, the mRS score was 1, NIHSS score was 6, ASPECTS was 8, and the mismatch ratio was >1.8. Therefore, after fully communicating with his family, we performed cerebrovascular interventional treatment for the proband. Cerebral angiography revealed a significant stenosis in the initial segment of the left MCA, measuring at 80%, with a blood flow graded at 2a (Figure 2B). Consequently, we carried out transcatheter intracranial vascular thrombectomy and conducted percutaneous balloon dilation on the left MCA. Following the intervention, there was a significant improvement in the previously observed stenosis. The residual stenosis was reduced to less than 20%, accompanied by a notable advancement in blood flow, graded at 3 (Figure 2C).

Intravenous antiplatelet therapy with tirofiban was continued for 48 h post-surgery, followed by the administration of oral dual antiplatelet medications, including aspirin and clopidogrel. No obvious bleeding lesions were found in the dynamic review of head CT 2 days after the operation (Supplementary Figure S5), and no vascular restenosis or hyperperfusion syndrome was found in the TCD review for three consecutive days after the operation. The laboratory tests indicated that LDL-C measured 2.81 mmol/L, homocysteine was 2.1 μmol/L, HbA1c stood at 6.2%, and high-sensitivity C-reactive protein was 11.9 mg/L. On the third day post-surgery, a comprehensive head magnetic resonance (MR) examination was conducted, and the DWI sequence unveiled the presence of multiple cerebral infarctions in the left frontotemporal parietal occipital lobe, basal ganglia, and right cerebellum (Figure 2D). Furthermore, the T2 FLAIR sequence showed more significant demyelinating changes in various regions compared to February (Figure 2E). Additionally, the T2 star-weighted angiography (SWAN) sequence indicated schistose hemorrhagic lesions in the parietal and occipital lobes (Figure 2F) and scattered microbleeds situated in the left frontal temporal parietal occipital lobe (Supplementary Figure S6). Over a span of approximately 13 days of treatment and rehabilitative care, there was a notable improvement in the symptoms of the proband.

**Figure 3:**
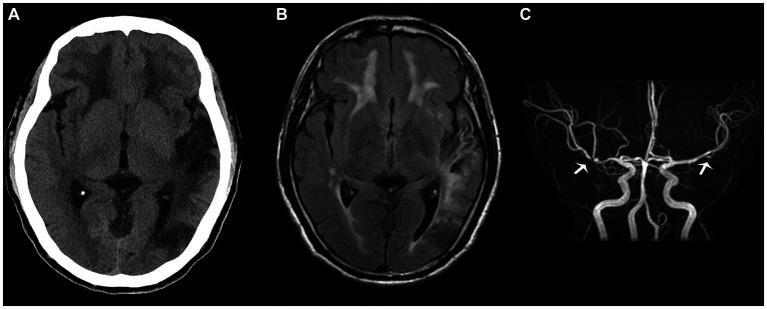
The proband’s head computed tomography re-examination in July **(A)** shows that the density of the left occipital and temporal lobes was lower than before, suggesting the formation of softening lesions; the T2 fluid attenuated inversion recovery sequence **(B)** in October shows multiple demyelinating changes in the white matter of the brain, while the magnetic resonance angiography sequence **(C)** depicts focal stenosis of the second segment of the left middle cerebral artery (MCA) and the first segment of the right MCA (white arrow).

However, upon discharge, physical examination indicated lingering signs of memory loss, speech ambiguity, and mixed and naming aphasia. Directional and computational abilities remained unaffected, while the right lower limb muscle strength was graded at V-. The limbs’ feeling was symmetrical, and the right Babinski sign remained positive. The NIHSS score was 2, the mRS score was 1, and the MOCA score stood at 12. Given the occlusion of M2 and the history of angioplasty treatment, the patient was advised to continue taking dual antiplatelet drugs orally for the next 3 months post-discharge, after which he should transition to monotherapy with antiplatelet medication. Symptomatic treatments, including cognitive improvement, pain relief, and lipid-lowering therapy, should also be maintained. Regular follow-up visits at the outpatient clinic were strongly recommended. So far, the proband has had head CT (Figure 3A) and head MR re-examinations (Figures 3B,C) in July and October, respectively. No intracranial hemorrhage or progression of demyelinating lesions has been observed, and his muscle strength has fully recovered. Only mixed aphasia and cognitive impairment remain. We have also developed a timeline detailing the proband’s progression, available in the Supplementary Material (Supplementary Figure S7).

## 3 Discussion

CADASIL is a prevalent hereditary stroke disease caused by mutations in the *NOTCH3* gene (17), typically expressed in VSMCs and pericytes (18). Degeneration and apoptosis of VSMCs and pericytes occur, resulting in impaired cerebrovascular reactivity and disruption of the blood–brain barrier (19, 20). Arterial lesions resulting from this gene mutation are systemic; however, small penetrating cerebral and leptomeningeal arteries are frequently the most severely impacted (2). This leads to strokes caused by CADASIL primarily involving lacunar infarcts in the subcortical white matter (21).

Our proband developed a large cerebral infarction due to occlusion of the left M2. We compiled research on various potential mechanisms underlying this M2 occlusion and subsequent cerebral infarction. Firstly, we considered the possibility of AS. The proband had a history of smoking, although diabetes was not diagnosed, the HbA1c level was elevated, and the color Doppler ultrasound of the neck vessels revealed bilateral carotid AS. Some studies have clarified that if patients with CADASIL experience the risk factors of AS, these risk factors will significantly accelerate the progress of the AS. This is because CADASIL with disconnection of vascular smooth muscle or pericytes allows the passage and accumulation of excessively secreted extracellular substances, leading to significant narrowing of the vessel wall (22, 23), which might be one of the reasons for the severe occlusion of the M2 in the proband. Additionally, we cannot dismiss the possibility of cardioembolism. Apart from the infarction observed in the left cerebral hemisphere, the proband also exhibited multiple infarcts involving both the anterior and posterior circulation, such as those seen in the left basal ganglia and right cerebellum. However, the dynamic electrocardiogram examination revealed no significant abnormalities, and the morphology of the blood vessels observed on his angiography suggested vascular stenosis rather than embolism. Furthermore, we consider whether it is attributable to CADASIL. Historically, CADASIL was believed to lack risk factors for macrovascular disease (24, 25). However, in recent years, there has been a growing number of reports and related clinical studies on CADASIL involving intracranial large vessel lesions. For instance, Kang and Kim enrolled 49 Korean patients with CADASIL, among whom 12 patients (24.5%) exhibited large intracranial artery stenosis or occlusion (26). Zhang et al. recruited 37 Chinese patients with CADASIL, with 28 individuals (75.7%) presenting intracranial large artery lesions (27). Nevertheless, it remains exceedingly rare for patients with CADASIL to undergo extensive cerebral infarction due to occlusion of large intracranial blood vessels. Given the mechanism of CADASIL vasculopathy, it is primarily characterized by impaired vascular reactivity and is generally not affecting lumen diameter (3, 7, 22). Therefore, we believe that the possibility of left M2 occlusion only caused by CADASIL is not significant.

However, regardless of the mechanism causing M2 segment obstruction, the treatment plan for the extensive area of cerebral infarction is consistent. According to the *Chinese Guideline for Endovascular Treatment of Acute Ischemic Stroke 2023*, thrombectomy can offer potential benefits for patients with moderate vascular occlusion, such as those affecting the M2 segment. Though, a careful evaluation must be made, considering both the patient’s overall condition and the complexity of the surgical procedure. The proband was admitted within 6 h of onset, and his mRS, NIHSS, and ASPECTS, along with the CTP results, all met the criteria for mechanical thrombectomy. Therefore, we performed an endovascular thrombectomy for the proband. It is worth noting that we have not encountered any reports on vascular interventional treatment for patients with CADASIL. Subsequently, venous antiplatelet therapy was initiated. Afterward, according to the guideline for the secondary prevention of IS (28), our plan was to transition to aspirin antiplatelet therapy following a 3 month course of oral dual antiplatelet drugs. However, the pathogenesis of CADASIL have indicated that antiplatelet therapy may exacerbate the incidence of cerebral microbleed lesions, thereby potentially elevating the risk of intra-cerebral hemorrhage (ICH) (29). Though, current evidence does not substantiate a clear link between the occurrence of ICH and the usage of antiplatelet drugs. According to reports, Muppa et al. conducted a clinical study on whether the use of antiplatelet drugs affected the incidence of AIS or ICH in patients with CADASIL (17). They included 455 patients with CADASIL and obtained negative results, indicating no significant difference in AIS or ICH events after receiving antiplatelet drug treatment. Furthermore, Choi et al. observed 62 patients diagnosed with CADASIL who were prescribed with antiplatelet drugs. They found no significant difference in the history of antiplatelet use between patients with ICH and those without (30). Nevertheless, the sample sizes included in these trials were relatively small. Therefore, to further elucidate the benefits and risks of antiplatelet drugs in patients with CADASIL, more prospective longitudinal studies and randomized controlled trials are necessary. In conclusion, based on the status of the proband at the time, we believe that using antiplatelet therapy was necessary.

After the discharge of the proband, considering the complexity of his condition and treatment plan, it is imperative to conduct regular outpatient follow-up visits to monitor their subsequent recovery and assess the risk of intracranial hemorrhage. For the sake of compliance and economic considerations for the proband, and compared with microbleeds, we are particularly concerned about the potential for symptomatic intracranial hemorrhage arising from the use of dual antiplatelet drugs. Therefore, we will mainly use head CT instead of SWAN sequence to monitor any intracranial hemorrhage in the future. So far, the proband has not experienced any intracranial hemorrhage, and the recovery status is favorable.

## 4 Conclusion

While cases with CADASIL primarily involve small intracranial blood vessels, there might be rare instances of large-area cerebral infarction due to occlusion of non-small vessels. There is a potential to enhance the outcome of patients by implementing endovascular procedures and antiplatelet drug treatments, along with consistent follow-up observations.

## Data availability statement

The datasets presented in this article are not readily available because of ethical and privacy restrictions. Requests to access the datasets should be directed to the corresponding authors.

## Ethics statement

Ethical review and approval was not required for the study on human participants in accordance with the local legislation and institutional requirements. Written informed consent from the patients/participants or patients/participants’ legal guardian/next of kin was not required to participate in this study in accordance with the national legislation and the institutional requirements. Written informed consent was obtained from the individual(s) for the publication of any potentially identifiable images or data included in this article.

## Author contributions

SX: Writing – review & editing, Writing – original draft, Validation, Methodology, Investigation, Conceptualization. MK: Writing – review & editing, Visualization, Supervision. KC: Writing – review & editing, Visualization. AX: Writing – review & editing, Supervision, Resources, Funding acquisition. MC: Writing – review & editing, Writing – original draft, Supervision, Methodology, Investigation, Conceptualization.
