# Supplementary material for: Treatment options for patients with CADASIL and large-scale cerebral infarction: mechanical thrombectomy and antiplatelet therapy—A case report
Source: Front Neurol. 2024 Jun 19;15:1400537. doi: 10.3389/fneur.2024.1400537 (PMC11221192; doi:10.3389/fneur.2024.1400537)
Supplement: Supplementary file 1 [file Data_Sheet_1.docx]

Supplementary Material


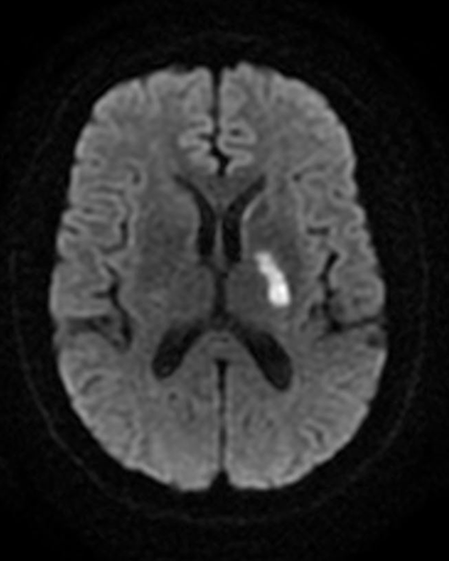


**Supplementary Figure 1.** The diffusion-weighted imaging sequence of the head magnetic resonance imaging of patient’s younger brother reveals a cerebral infarction in the left basal ganglia-corona radiata area.


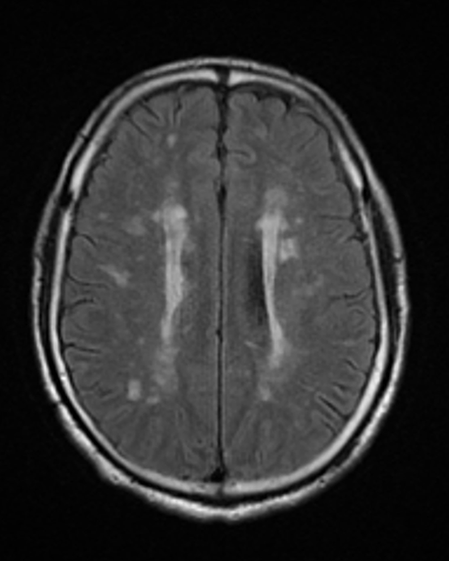


**Supplementary Figure 2.** The T2 fluid attenuated inversion recovery sequence of patient’s younger brother shows demyelinating changes in the white matter of central oval, and radiocoronal regions around the bilateral lateral ventricles.


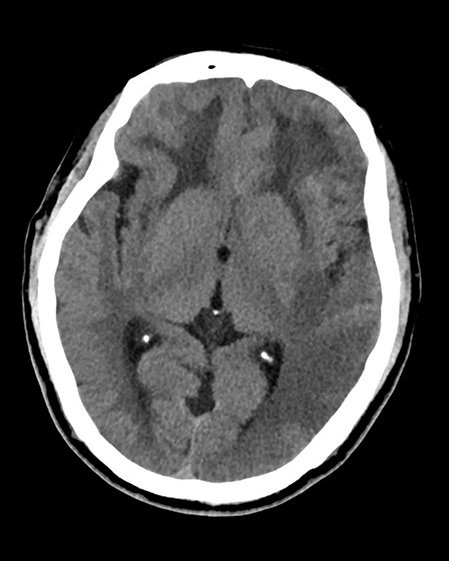


**Supplementary Figure 3.** The emergency head computed tomography of the proband reveals an extensive cerebral infarction in the left temporal parietal occipital lobe.


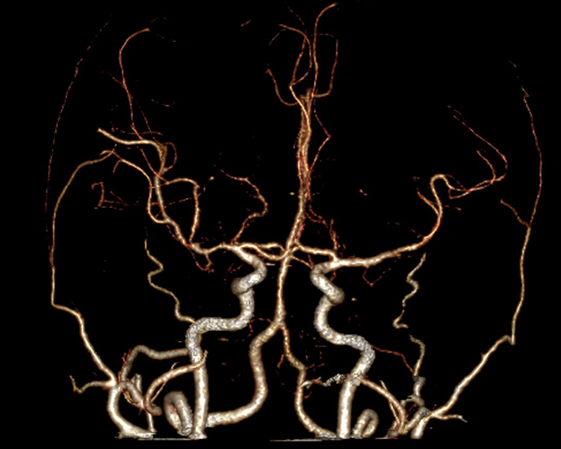


**Supplementary Figure 4.** The emergency head computer tomography angiography of the proband discloses occlusion of the distal lumen in the left of the second middle cerebral artery (MCA) segment and local stenosis of the right of the first MCA segment.


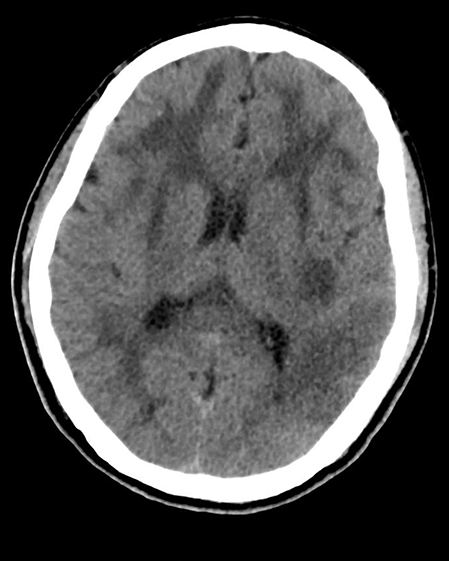


**Supplementary Figure 5.** The head computed tomography after the operation reveals a low density shadow in the left temporal parietal lobe with brain tissue swelling.


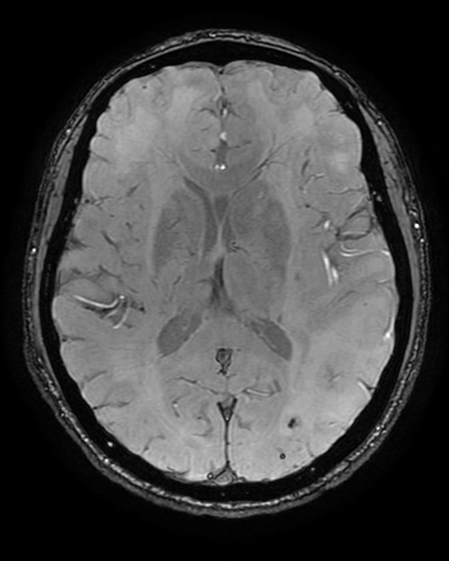


**Supplementary Figure 6.** The T2 star-weighted angiography sequence indicates scattered microbleeds in the left occipital lobe.


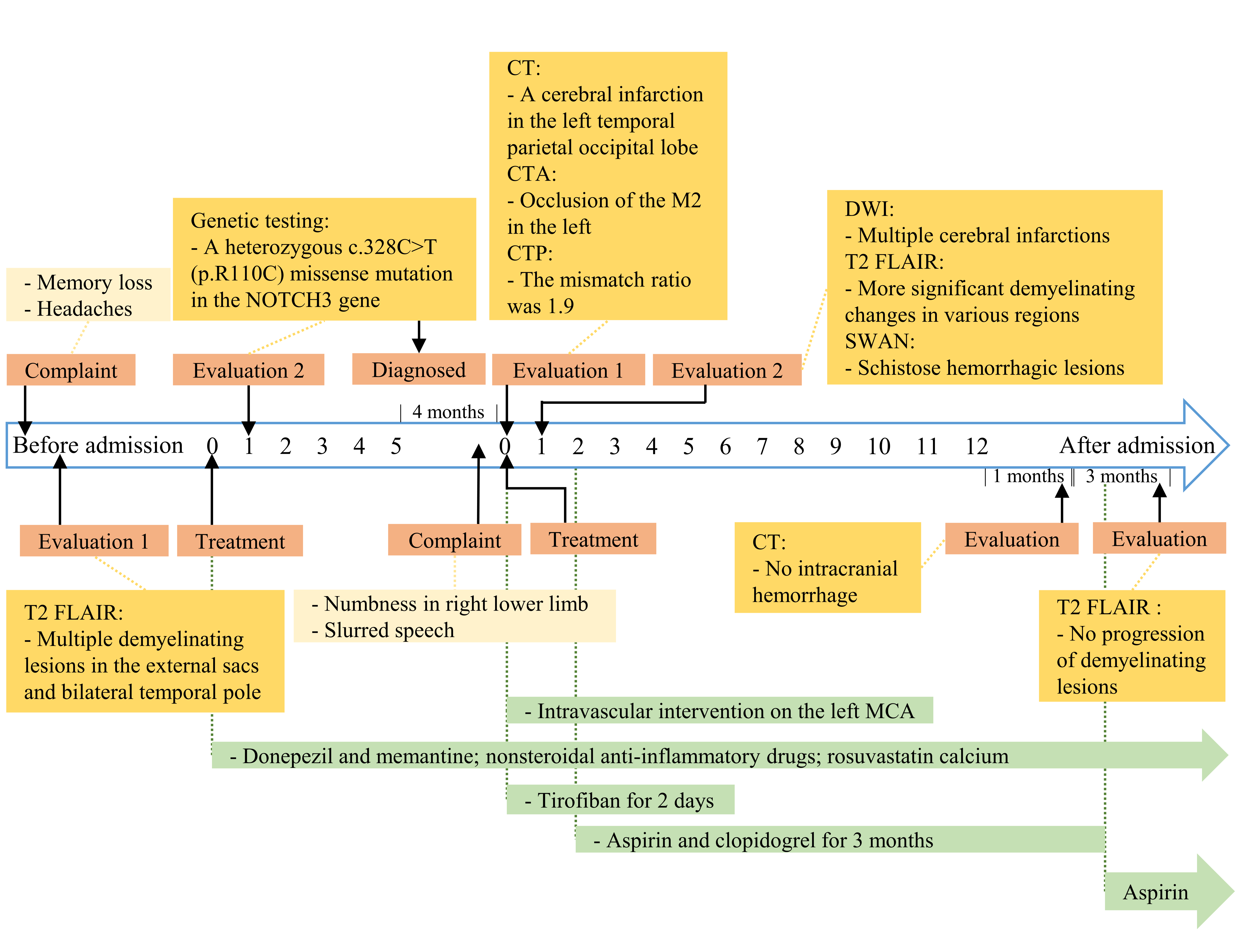


**Supplementary Figure 7.** Timeline displaying the evolution of this case: in the white box, the dates have been reported; in the orange-red boxes, important events was listed; in the pale yellow boxes, symptoms was described; in the green boxes, therapies was conducted; and in the golden yellow boxes, we have displayed the evaluations. CT, computed tomography; CTA, computer tomography angiography; CTP, computer tomography perfusion; DWI, diffusion-weighted imaging; MCA, middle cerebral artery; SWAN, T2 star-weighted angiography; T2 FLAIR, T2 fluid attenuated inversion recovery.
